# On the transmission dynamics of Buruli ulcer in Ghana: Insights through a mathematical model

**DOI:** 10.1186/s13104-015-1619-5

**Published:** 2015-11-06

**Authors:** Farai Nyabadza, Ebenezer Bonyah

**Affiliations:** Department of Mathematical Sciences, Stellenbosch University, Private Bag X1, Matieland, 7602 South Africa; Department of Mathematics and Statistics, Kumasi Polytechnic, P. O. Box 854, Kumasi, Ghana

**Keywords:** Buruli ulcer, Transmission dynamics, Basic reproduction number, Sensitivity analysis, Stability

## Abstract

**Background:**

*Mycobacterium ulcerans* is know to cause the Buruli ulcer. The association between the ulcer and environmental exposure has been documented. However, the epidemiology of the ulcer is not well understood. A hypothesised transmission involves humans being bitten by the water bugs that prey on mollusks, snails and young fishes.

**Methods:**

In this paper, a model for the transmission of *Mycobacterium ulcerans* to humans in the presence of a preventive strategy is proposed and analysed. The model equilibria are determined and conditions for the existence of the equilibria established. The model analysis is carried out in terms of the reproduction number $\mathcal{R}_0$. The disease free equilibrium is found to be locally asymptotically stable for $\mathcal{R}_0<1.$ The model is fitted to data from Ghana.

**Results:**

The model is found to exhibit a backward bifurcation and the endemic equilibrium point is globally stable when $\mathcal{R}_0>1.$ Sensitivity analysis showed that the Buruli ulcer epidemic is highly influenced by the shedding and clearance rates of *Mycobacterium ulcerans* in the environment. The model is found to fit reasonably well to data from Ghana and projections on the future of the Buruli ulcer epidemic are also made.

**Conclusions:**

The model reasonably fitted data from Ghana. The fitting process showed data that appeared to have reached a steady state and projections showed that the epidemic levels will remain the same for the projected time. The implications of the results to policy and future management of the disease are discussed.

## Background

Buruli ulcer is caused by pathogenic bacterium where infection often leads to extensive destruction of skin and soft tissue through the formation of large ulcers usually on the legs or arms [28]. It is a devastating disease caused by *Mycobacterium ulcerans*. The ulcer is fast becoming a debilitating affliction in many countries [3]. It is named after a region called Buruli, near the Nile River in Uganda, where in 1961 the first large number of cases was reported. In Africa, close to 30,000 cases were reported between 2005 and 2010 [29]. Cote d’Ivoire, with the highest incidence, reported 2533 cases in 2010 [27]. This disease has dramatically emerged in several west African countries, such as Ghana, Cote d’Ivoire, Benin, and Togo in recent years [26].

The transmission mode of the ulcer is not well understood, however residence near an aquatic environment has been identified as a risk factor for the ulcer in Africa [6, 16, 25]. Transmission is thus likely to occur through contact with the environment [20]. Recent studies in West Africa have implicated aquatic bugs as transmission vectors for the ulcer [18, 24]. An attractive hypothesis for a possible mode of transmission to humans was proposed by Portaels et al. [22]: water-filtering hosts (fish, mollusks) concentrate the *Mycobacterium ulcerans* bacteria present in water or mud and discharge them again to this environment, where they are then ingested by aquatic predators such as beetles and water bugs. These insects, in turn, may transmit the disease to humans by biting [18]. Person to person transmission is less likely. Aquatic bugs are insects found throughout temperate and tropical environments with abundant freshwater. They prey, according to their size, on mollusks, snails, young fishes, and the adults and larvae of other insects that they capture with their raptorial front legs and bite with their rostrum. These insects can inflict painful bites on humans as well. In Ghana, where Buruli ulcer is endemic, the water bugs are present in swamps and rivers, where human activities such as farming, fishing, and bathing take place [18].

Research on Buruli ulcer has focused mainly on the socio-cultural aspects of the disease. The research recommends the need for Information, Education and Communication (IEC) intervention strategies, to encourage early case detection and treatment with the assumption that once people gain knowledge they will take the appropriate action to access treatment early [2]. IEC is defined as an approach which attempts to change or reinforce a set of behaviours to a targeted group regarding a problem. The IEC strategy is preventive in that it has a potential of enhancing control of the ulcer [5]. It is also important to note that Buruli ulcer is treatable with antibiotics. A combination of rifampin and streptomycin administered daily for 8 weeks has the potential to eliminate *Mycobacterium ulcerans* bacilli and promote healing without relapse.

Mathematical models have been used to model the transmission of many diseases globally. Many advances in the management of diseases have been born from mathematical modeling [11, 12, 14, 15]. Mathematical models can evaluate actual or potential control measures in the absence of experiments, see for instance [19]. To the best of our knowledge very few mathematical models have been formulated to analyse the transmission dynamics of Mycobacterium ulcerans. This could be largely due to the elusive epidemiology of the Buruli ulcer. Aidoo and Osei [3] proposed a mathematical model of the *SIR*-type in an endeavour to explain the transmission of *Mycobacterium ulcerans* and its dependence on arsenic. In this paper, we propose a model which takes into account the human population, water bugs as vectors and fish as potential reservoirs of *Mycobacterium ulcerans* following the transmission dynamics described in [8]. In addition we include the preventive control measures in a bid to capture the IEC strategy. Our main aim is to study the dynamics of the Buruli ulcer in the presence of a preventive control strategy, while emphasizing the role of the vector (water bugs) and fish and their interaction with the environment. The model is then validated using data from Ghana. This is crucial in informing policy and suggesting strategies for the control of the disease.

This paper is arranged as follows; in “Methods”, we formulate and establish the basic properties of the model. We also determine the steady states and analysed their stability. The results of this paper are given in “Results”. Parameter estimation, sensitivity analysis and the numerical results on the behavior of the model are also presented in this section. The paper is concluded in “Discussion”.

## Methods

### Model formulation

We consider a constant human population $N_H(t),$ the vector population of water bugs $N_V(t)$ and the fish population $N_F(t)$ at any time *t*. The total human population is divided into three epidemiological subclasses of those that are susceptible $S_H(t),$ the infected $I_H(t)$ and the recovered who are still immune $R_H(t)$. Total population of vector (water bug) at any time *t* is divided into two subclasses to susceptible water bugs $S_V(t)$ and those that are infectious and can transmit the Buruli ulcer to humans, $I_V(t).$ The total population reservoir of small fish is also divided into two compartments of susceptible fish $S_F(t)$ and infected fish $I_F(t).$ We also consider the role of the environment by introducing a compartment *U*,  representing the density of *Mycobacterium ulcerans* in the environment. We make the following basic assumptions:

- *Mycobacterium ulcerans* are transferred only from vector ( water bug) to the humans.
- There is homogeneity of human, water bug and fish populations’ interactions.
- Infected humans recover and are temporarily immune, but lose immunity.
- Fish are preyed on by the water bugs.
- Unlike some bacterial infections such as leprosy (caused by *Mycobacterium leprae*) and tuberculosis (caused by *Mycobacterium tuberculosis*), which are characterized by person-to-person contact transmission, it is hypothesized that *Mycobacterium ulcerans* is acquired through environmental contact and direct person-to-person transmission is rare [20].
- Susceptible host (human population) can be infected through biting by an infectious vector (water bug). We represent the effective biting rate that an infectious vector has to susceptible host as $\beta _H$ and the incidence of new infections transmitted by water bugs is expressed by standard incidence rate $\displaystyle \beta _H \frac{S_H I_V}{N_H}.$ One can interpret $\beta _H$ as a function of the biting frequency of the infected water bugs on humans, density of infectious water bugs per human, the probability that a bite will result in an infection and the efficacy of the IEC strategy. In particular we can set $\beta _H=(1-\epsilon )\tau \alpha \beta _H^*,$ where $\epsilon \in (0,1)$ is the efficacy of the IEC strategy, $\tau$ the number of water bugs per human host, $\alpha$ the biting frequency (the biting rate of humans by a single water bug) and $\beta _H^*$ the probability that a bite by an infected vector to a susceptible human will produce an infection.
- Susceptible water bugs are infected at a rate $\displaystyle \beta _V \frac{S_V I_F}{N_V}$ through predation of infected fish and $\displaystyle \eta _v\beta _V \frac{S_V U}{K}$ representing other sources in the environment. Here $\eta _V$ differentiates the infectivity potential of the fish from that of the environment.
- Assuming fish prey on infected water bugs, susceptible fish are infected at a rate $\displaystyle \beta _F\frac{S_F I_V}{N_F}$ through predation of infected fish and $\displaystyle \eta _F\beta _F \frac{S_F U}{K}$ representing infection through the environment. Here $\eta _F$ is a modification parameter that models the relative infectivity of fish from that of the environment.
- The vector population and the fish populations are assumed to be constant. The growth functions are respectively given by $g(N_V)$ and $g(N_F),$ where
  $$\begin{aligned} g(N_V)=\mu _VN_V~~\mathrm{and}~~g(N_F)=\mu _FN_F. \end{aligned}$$
  It is important to note that other types of functions can be chosen as growth functions. In this work we however assume that the growth functions are linear.
- There is a proposed hypothesis that environmental mycobacteria in the bottoms of swamps may be mechanically concentrated by small water-filtering organisms such as microphagous fish, snails, mosquito larvae, small crustaceans, and protozoa [8]. We assume that fish increase the environmental concentrations of *Mycobacterium ulcerans* at a rate $\sigma _F.$ Humans are are assumed not to shed any bacteria into the environment.
- Aquatic bugs release bacteria into the environment at a rate $\sigma _V.$
- The model does not include a potential route of direct contact with the bacterium in water.
- The birth rate of the human population is directly proportional to the size of the human population.
- The recovery of infected individuals is assumed to occur both spontaneously and through treatment. Research has shown that localized lesions may spontaneously heal but, without treatment, most cases of Buruli ulcer result in physical deformities that often lead to physiological abnormalities and stigmas [4].

**Fig. 1:**
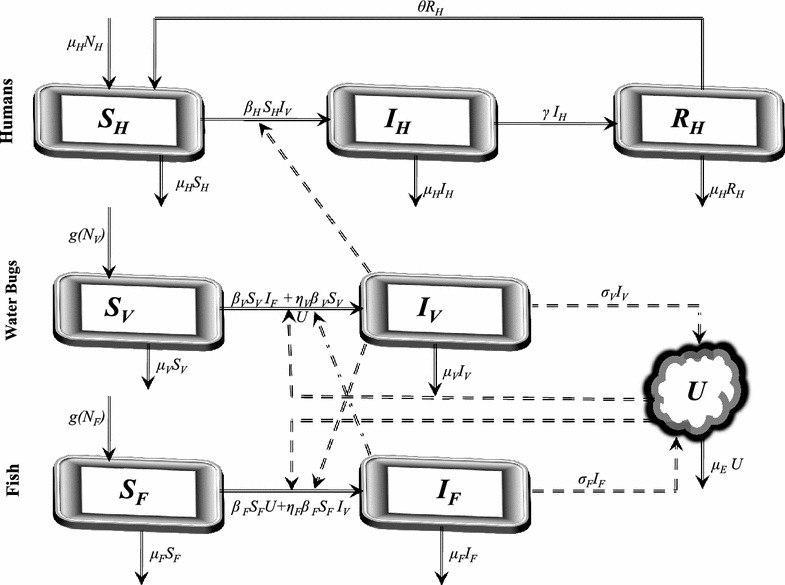
Proposed transmission dynamics of the Buruli ulcer among humans, fish, water bugs and the environment (*U*)

We now describe briefly, the transmission dynamics of Buruli ulcer:

New susceptibles enter the population at a rate of $\mu _H N_H.$ Buruli ulcer sufferers do not recover with permanent immunity, they loose immunity at a rate $\theta$ and become susceptible again. Susceptibles and infected through interaction with infected water bugs, with infection driven by water bugs biting susceptible humans. Once infected, individuals are allowed to recover either spontaneously or through antibiotic treatment at a rate $\gamma .$ In this model, the human population is assumed to be constant over the modeling time with the birth and death rates being equal. The compartment $S_V$ tracks the changes in the susceptible water bugs population that are recruited at a rate $\mu _V N_V$. The infection of water bugs is driven by two processes: their interaction infected fish and with the environment. The natural mortality of the water bugs occurs at a rate $\mu _V.$ Similarly, the compartment $S_F$ tracks the changes in the susceptible fish population that are recruited at a rate $\mu _FN_F$. The infection of fish is also driven by two processes: their interaction infected water bugs and with the environment. Fish’s natural mortality rate is $\mu _F.$ The growth of *Mycobacterium ulcerans* in the environment is driven by their shedding by infected water bugs and fish into the environment. They are assumed to die naturally at a rate $\mu _E.$ The possible interrelations between humans, the water bug and fish are represented by the schematic diagram below (Fig. 1).

The descriptions of the parameters that describe the flow rates between compartments are given in Table 1.

**Table 1:** Description of parameters used in the model

Symbol	Description
$\beta _H$	The effective contact rate between the vector and susceptible humans
$\beta _V$	The effective contact rate between fish and susceptible vectors
$\beta _F$	The effective contact rate between the susceptible fish and *Mycobacterium ulcerans*
$\gamma$	The recovery rate of infected humans
$\theta$	The rate of loss of immunity of recovered humans
$\mu _H$	Natural mortality rate/birth rate of the human population
$\mu _V$	Natural mortality rate of the vector population
$\mu _F$	Natural mortality rate of the fish population
$r_V$	The growth rate of the vector population
$r_F$	The growth rate of the fish population
*K*	The environmental carrying capacity of the bacteria population
$\sigma _F$	Rate of shedding of *Mycobacterium ulcerans* into the environment by fish
$\sigma _V$	Rate of shedding of *Mycobacterium ulcerans* into the environment by the water bugs
$\mu _E$	Rate at which *Mycobacterium ulcerans* are cleared from the environment

The dynamics of the ulcer can be described by the following set of nonlinear differential equations:

1 $$\begin{aligned} \left. \begin{array}{lcl} \displaystyle \frac{dS_H}{dt}&{}= &{} \displaystyle \mu _HN_H +\theta R_H - \beta _H\frac{S_HI_V}{N_H}-{\mu _H}S_H,\\ \displaystyle \frac{dI_H}{dt}&{} = &{} \displaystyle \beta _H\frac{S_HI_V}{N_H} - ({\mu _H} +\gamma )I_H,\\ \displaystyle \frac{dR_H}{dt}&{} =&{} \displaystyle \gamma I_H-(\mu _H+\theta )R_H,\\ \displaystyle \frac{dS_V}{dt}&{} = &{}\displaystyle \mu _VN_V -\beta _V\frac{S_VI_F}{N_V}-\eta _V\beta _V\frac{S_VU}{K}- {\mu _V}S_V,\\ \displaystyle \frac{dI_V}{dt}&{} = &{} \displaystyle \beta _V\frac{S_VI_F}{N_V}+\eta _V\beta _V\frac{S_VU}{K}- {\mu _V}I_V,\\ \displaystyle \frac{dS_F}{dt}&{} = &{} \displaystyle \mu _FN_F-\beta _F\frac{S_FI_V}{N_F} -\eta _F\beta _F\frac{S_FU}{K}- {\mu _F}S_F,\\ \displaystyle \frac{dI_F}{dt}&{} = &{} \displaystyle \beta _F\frac{S_FI_V}{N_F}+\eta _F\beta _F\frac{S_FU}{K}- {\mu _F}I_F,\\ \displaystyle \frac{dU}{dt}&{} = &{} \displaystyle \sigma _FI_F+\sigma _VI_V- {\mu _E}U. \end{array} \right\} \end{aligned}$$

We assume that all the model parameters are positive and the initial conditions of the model system (1) are given by

$$\begin{aligned} S_H(0)&=  {} S_{H0} > 0, I_H(0) = I_{H0}\ge 0, R_H(0)= R_{H0}= 0,~S_V(0) = S_{V0} > 0,\\ I_V(0)&=  {} I_{V0}\ge 0,~S_F(0) = S_{F0} > 0, ~I_F(0) = I_{F0}\ge 0 \quad \text {and}\quad U(0)=U_0>0. \end{aligned}$$

We arbitrarily scale the time *t* by the quantity ${1 \over {\mu _V }}$ by letting $\tau = \mu _Vt$ and introduce the following dimensionless parameters;

$$\begin{aligned} \tau&=  {} \mu _Vt,~ \beta _h=\frac{\beta _H}{\mu _V},~\mu _h=\frac{\mu _H}{\mu _V},~ \theta _h=\frac{\theta }{\mu _V}, ~\gamma _h=\frac{\gamma }{\mu _V}, ~m_1=\frac{N_H}{N_V},~m_2=\frac{N_F}{N_V},\\ m_3&= {} \frac{1}{m_2},~m_4=\frac{N_F}{K},~m_5=\frac{N_V}{K},~ \mu _f=\frac{\mu _F}{\mu _V},~\beta _f=\frac{\beta _F}{\mu _V},\\ \sigma _f&= {} \frac{\sigma _F}{\mu _V},~\sigma _v=\frac{\sigma _V}{\mu _V},~\beta _v=\frac{\beta _V}{\mu _V} \;\mathrm{and}\;\mu _e=\frac{\mu _E}{\mu _V}. \end{aligned}$$

can be non dimensionalised bySo, system (1) setting

$$\begin{aligned} s_h=\frac{S_H}{N_H},~i_h=\frac{I_H}{N_H},r_h=\frac{R_H}{N_H},~i_v=\frac{I_V}{N_V},~s_f=\frac{S_F}{N_F},~i_f=\frac{I_F}{N_F}\;\mathrm{and}\;\displaystyle u=\frac{U}{K}. \end{aligned}$$

The forces of infection for humans, water bugs and fish are respectively

$$\begin{aligned} \lambda _H=\beta _h m_1i_v,~~\lambda _V=\beta _v m_2i_f+\eta _V\beta _v u,~~\lambda _F=\beta _f m_3i_v+\eta _F\beta _f u. \end{aligned}$$

Given that the total number of bites made by the water bugs must equal the number of bites received by the humans, $m_1$ is a constant, see [9]. Similarly $m_2$ is constant and so is $m_3.$ We also note that since $N_F$ and $N_V$ are constants, $m_4$ and $m_5$ are constants.

Given that $\displaystyle s_h+i_h+r_h=1,~s_v+i_v=1,~s_f+i_f=1$ and $\displaystyle 0\le u\le 1,$ system (1) can be reduced to the following system of equations by conveniently maintaining the capitalised subscripts so that we can still respectively write $\displaystyle s_h,~i_h,~i_v,~i_f$ and $\displaystyle u$ as $\displaystyle S_H,~I_H,~I_V,~I_F$ and $\displaystyle U.$

2 $$\begin{aligned} \left. \begin{array}{lcl} \displaystyle \frac{dS_H}{d\tau }&{}= &{} \displaystyle (\mu _h +\theta _h)(1- S_H) -\theta _h I_H - \lambda _H S_H,\\ \\ \displaystyle \frac{dI_H}{d\tau }&{} = &{} \displaystyle \lambda _HS_H - ({\mu _h} +\gamma _h)I_H,\\ \\ \displaystyle \frac{dI_V}{d\tau }&{} = &{} \displaystyle \lambda _V(1-I_V)-\mu _vI_V,\\ \\ \displaystyle \frac{dI_F}{d\tau }&{} = &{} \displaystyle \lambda _F(1-I_F)- {\mu _f}I_F,\\ \\ \displaystyle \frac{dU}{d\tau }&{} = &{} \displaystyle m_4\sigma _f I_F+m_5\sigma _vI_V- {\mu _e}U. \end{array} \right\} \end{aligned}$$

### Basic properties

#### Feasible region

Note that $\displaystyle \frac{dU}{d\tau }=m_4\sigma _f I_F+m_5\sigma _vI_V- {\mu _e}U\le m_4\sigma _f +\,m_5\sigma _v-\mu _eU.$ Through integration we obtain $\displaystyle U\le \frac{m_4\sigma _f +m_5\sigma _v}{\mu _e}.$ The feasible region (the region where the model makes biological sense) for the system (2) is in $\mathbb {R}^5_+$ and is represented by the set

$$\begin{aligned} \Omega= & {} \left\{ (S_H,I_H,I_V,I_F,U)\in \mathbb {R}^5_+|0\le S_H+I_H\le 1,0\le I_V\le 1, 0\le I_F\le 1,\right. \\&~~~\left. 0\le U\le \frac{m_4\sigma _f +m_5\sigma _v}{\mu _e}\right\} , \end{aligned}$$

where the basic properties of local existence, uniqueness and continuity of solutions are valid for the Lipschitzian system (2). The populations described in this model are assumed to be constant over the modelling time. The solutions of system (2) starting in $\displaystyle \Omega$ remain in $\displaystyle \Omega$ for all $t>0.$ Thus , $\displaystyle \Omega$ is positively invariant and it is sufficient to consider solutions in $\displaystyle \Omega .$

#### Positivity of solutions

We desire to show that for any non-negative initial conditions of system (2), say $\displaystyle (S_{H0},I_{H0},I_{V0},I_{F0},U_0),$ the solutions remain non-negative for all $\displaystyle \tau \in [0,\infty ).$ We prove that all the state variables remain non-negative and the solutions of the system (2) with positive initial conditions will remain positive for all $\tau > 0$. We thus state the following lemma.

##### **Lemma 1**

*Given that the initial conditions of system* (2) *are positive, the solutions*$S_H(\tau ),~I_H(\tau ),~I_V(\tau ),~I_F(\tau )$*and*$U(\tau )$*are non-negative for all*$\tau >0$.

##### *Proof*

Assume that

$$\begin{aligned} \hat{\tau } = \sup \left\{ \tau >0: S_H>0, I_H>0, I_V>0, I_F>0, U >0\right\} \in ( 0, \tau ]. \end{aligned}$$

Thus $\hat{\tau } > 0,$ and it follows directly from the first equation of the system (2) that

$$\begin{aligned} \frac{dS_H}{d\tau } \ge - (\theta _h + \lambda _H)S_H. \end{aligned}$$

We thus have

$$\begin{aligned} \frac{dS_H}{dt}\ge S_{H0}\exp \left[ - \theta _h t+ \int _0^\tau \lambda _H(\varsigma )d\varsigma \right] . \end{aligned}$$

Since the exponential function is always positive and $S_{H0}=S_H(0)>0,$ the solution $S_H(\tau )$ will thus be always positive.

From the second equation of (2),

$$\begin{aligned} \frac{dI_H}{d\tau }&\ge -(\mu _h+\gamma _h)I_H,\\ \Rightarrow I_H&\ge I_{H0}e^{-(\mu _h +\gamma _h)\tau }>0. \end{aligned}$$

Similarly, it can be shown that $I_V(\tau ) > 0,~I_F(\tau ) > 0$ and $U(\tau ) > 0$ for all $\tau > 0 ,$ and this completes the proof. $\square$

### Steady states analysis

#### The disease free equilibrium

In this section, we solve for the equilibrium points by setting the right hand side of system (2) to zero. This direct calculation shows that system (2) always has a disease free equilibrium point

$$\begin{aligned} \mathbf{\mathcal {E}_0}=(1,0,0,0,0). \end{aligned}$$

We have the following result on the local stability of the disease free equilibrium.

##### **Theorem 1**

*The disease free equilibrium*$\mathbf{\mathcal {E}_0}$*whenever it exists, is locally asymptotically stable if*$\mathcal{R}_0 <1$*and unstable otherwise.*

##### *Proof*

The Jacobian matrix of system (2) at the equilibrium point $\mathbf{\mathcal {E}_0}$ is given by

$$\begin{aligned} J_{\mathbf{\mathcal {E}_0}}&= \left( \begin{array}{ccccc} -(\mu _h+\theta _h) &{}-\theta _h &{}-m_1\beta _h&{} 0&{}0 \\ 0&{} -(\mu _h+\gamma _h) &{}m_1\beta _h&{}0&{}0 \\ 0&{} 0 &{}-1&{}m_2\beta _v&{}\eta _v\beta _v\\ 0&{} 0 &{}m_3\beta _f&{}-\mu _f&{}\eta _f\beta _f\\ 0&{} 0 &{}m_5\sigma _v&{}m_4\sigma _f&{}-\mu _e \end{array} \right) . \end{aligned}$$

It can be seen that the eigenvalues of $\displaystyle J_{\mathbf{\mathcal {E}_0}}$ are $-(\mu _h+\theta _h),~ -(\mu _h+\gamma _h)$ and the solution of the characteristic polynomial

$$\begin{aligned} P(\vartheta )=\vartheta ^3+a_2\vartheta ^2 + a_1\vartheta +\mu _e\mu _f(1-\mathcal{R}_0)=0, \end{aligned}$$

where

$$\begin{aligned} a_2&=  {} 1+\mu _e+\mu _f,\\ a_1&=  {} \mu _e+\mu _f+\mu _e\mu _f-(\beta _f\beta _v+m_4\eta _f\sigma _f\beta _f+m_5\eta _v\sigma _v\beta _v)~~\mathrm{and}\\ \mathcal{R}_0&=  {} R_0^1+R_0^2+R_0^3, \end{aligned}$$

for

$$\begin{aligned} R_0^1=\frac{m_4\eta _f\sigma _f\beta _f}{\mu _e\mu _f},~R_0^2=\frac{m_5\eta _v\sigma _v\beta _v}{\mu _e} \quad \mathrm{and} \quad R_0^3=\beta _f\beta _v\left( \frac{\mu _e+m_3m_4\eta _v\sigma _f+m_2m_5\eta _f\sigma _v}{\mu _e\mu _f}\right) . \end{aligned}$$

The solutions of $$P(\vartheta )=0$$ have negative real parts only if $$\displaystyle \mathcal{R}_0<1$$ following the use of the Routh Hurwitz Criterion. We can thus conclude that the disease free equilibrium is locally asymptotically stable whenever $$\displaystyle \mathcal{R}_0<1.$$$$\square $$

We note that $\displaystyle \mathcal{R}_0$ is the model system (2)’s reproduction number and does not depend on the human population size. The model reproduction number is a sum of three terms. The terms $R_0^1$ and $R_0^2$ represent the contribution of fish and water bugs respectively to the infection dynamics. The term $R_0^3,$ which is not very common in many epidemiological models, shows the combined contribution of the water bugs, fish and their shedding of *Mycobacterium ulcerans* into the environment. So, the infection is driven by the water bugs, fish and the density of the bacterium in the environment. The model reproduction number increases linearly with the shedding rates of the *Mycobacterium ulcerans* into the environment by fish and water bugs and the effective contact rates $\beta _f$ and $\beta _v$. It decreases with increasing removal rates of the fish and *Mycobacterium ulcerans*. So the control of the ulcer depends largely on environmental management.

#### The endemic equilibrium

The endemic equilibrium is much more tedious to obtain. Given that $\displaystyle \lambda ^*_H=\beta _hm_1I_V^*,$ from the first and second equations of system (2) we have

$$\begin{aligned} S_H^*=\frac{1}{1+\mathcal{A}I_V^*} \quad \mathrm{and}\quad I_H^*=\frac{m_1\beta _hI_V^*}{(\mu _h+\gamma _h)(1+\mathcal{A}I_V^*)}, \end{aligned}$$

where $$\displaystyle \mathcal{A}=\frac{m_1\beta _h(\mu _h+\theta _h+\gamma _h)}{(\mu _h+\gamma _h)(\mu _h+\theta _h)}.$$

The last equation of system (2) can be written as

$$\begin{aligned} U^*=\vartheta _1I_F^*+\vartheta _2I_V^*, \quad \mathrm{where}~\vartheta _1=\frac{m_4\sigma _f}{\mu _e}~ \mathrm{and}~\vartheta _2=\frac{m_5\sigma _v}{\mu _e}. \end{aligned}$$

We thus have

$$\begin{aligned} \lambda ^*_F=\vartheta _3I_V^*+\vartheta _4I_F^ \mathrm{and}~\lambda ^*_V=\vartheta _5I_V^*+\vartheta _6I_F^*, \end{aligned}$$

where $$\displaystyle \vartheta _3=\beta _f(m_3+\vartheta _2\eta _f),~\vartheta _4=\vartheta _1\beta _f\eta _f,~\vartheta _5=\vartheta _2\eta _v\beta _v ~\mathrm{and}~\vartheta _6=\beta _v(m_2+\vartheta _1\eta _v).$$

From the third and fourth equations of system (2)we have

3 $$\begin{aligned} I_F^*=\, & {} \frac{I_V^*[1-\vartheta _5(1-I_V^*)]}{\vartheta _6(1-I_V^*)},\end{aligned}$$

4 $$\begin{aligned} I_V^*=\, & {} \frac{I_F^*[\mu _f-\vartheta _4(1-I_F^*)]}{\vartheta _3(1-I_F^*)}. \end{aligned}$$

Substituting (3) into (4) we obtain $\displaystyle I_V^*=0$ and the cubic equation

5 $$\begin{aligned} f(I_V^*)=a_3{I_V^*}^3+a_2{I_V^*}^2+a_1I_V^*+a_0=0, \end{aligned}$$

where

$$\begin{aligned} a_0&=  {} \frac{\beta _f\mu _f}{\mu _e}\left( \mu _em_2+m_4\eta _v\sigma _f\right) \left[ \mathcal{R}_0-1\right] ,\\ a_1&=  {} \vartheta _4\vartheta _5(1+\vartheta _6)+\vartheta _5(\vartheta _4+\vartheta _3\vartheta _6)+\vartheta _3\vartheta _5\vartheta _6-[\vartheta _3\vartheta _6(1+\vartheta _6)+ \vartheta _5(\vartheta _4\vartheta _5+\mu _f\vartheta _6)+\vartheta _4\vartheta _5^2],\\ a_2&=  {} (1+\vartheta _6)(\vartheta _4+\vartheta _3\vartheta _6)+\vartheta _5(\vartheta _4\vartheta _5+\mu _f\vartheta _6)+\vartheta _6(\vartheta _3\vartheta _6+\mu _f\vartheta _5)-[ 2\vartheta _4\vartheta _5(1+\vartheta _6)+\vartheta _6(\vartheta _3\vartheta _5+\mu _f)],\\ a_3&=  {} -\frac{m_5\beta _f\eta _v\sigma _v\beta _v^2}{\mu _e^2}\left( (\mu _em_2+m_4\eta _v\sigma _f)m_3+m_2m_5\eta _f\sigma _v\right) <0. \end{aligned}$$

Note that

$$\begin{aligned} a_0 \left\{ \begin{array}{ll}> 0\quad \mathrm{if}\quad \mathcal{R}_0>1\\ <0\quad \mathrm{if}\quad \mathcal{R}_0<1. \end{array}\right. \end{aligned}$$

Given that

6 $$\begin{aligned} f'(I_V^*) = 3a_3(I_V^*)^2 + 2a_2\lambda _1^* + a_1 , \end{aligned}$$

the turning points of equation (5) are given by

7 $$\begin{aligned} (I_V^*)^{1,2} = \dfrac{-a_2 \pm \sqrt{a_2^2 - 3 a_1a_3}}{3a_3}. \end{aligned}$$

The discriminant of solutions (7) is $\triangle = a_2^2 - 3 a_1a_3$. We now focus on the sign of the discriminant.

If $\triangle <0$, then $f(I_V^*)$ has no real turning points, which implies that $f(I_V^*)$ is a strictly monotonic function. The sign of $f'(\lambda _1^*)$ is crucial in determining the monotonicity. Through completing the square, equation (6) can be written as

8 $$\begin{aligned} f'(I_V^*) = 3a_3 \left[ \left( {I_V^*} + \dfrac{a_2}{3a_3} \right) ^2 + \dfrac{1}{9a_3^2}(3 a_1a_3-a_2^2) \right] . \end{aligned}$$

Clearly if $\triangle <0$, then $3 a_1a_3-a_2^2>0$. Since $a_3<0$, then $f'(I_V^*)<0$. Thus $f(I_V^*)$ is a strictly monotone decreasing function. Note that $\lim _{I_V^*\rightarrow \mp \infty } f(I_V^*)=\pm \infty$. For $f(0) = a_0<0,$ the polynomial $f(I_V^*)$ has no positive real roots for $\mathcal{R}_0<1,$. However, if $f(0) = a_0>0$ it has only one positive real root for $\mathcal{R}_0>1,$ and consequently only one endemic equilibrium.

If $\triangle =0$, then $f'(I_V^*)$ has only one real root with multiplicity two. This implies that $(I_V^*)^1=(I_V^*)^2 = -\frac{a_2}{3a_3}$ and that $f'(I_V^*)<0$. Thus the polynomial $f(I_V^*)$ is a decreasing function. Given that $f''(I_V^*)(-\frac{a_2}{3a_3}) = 0,$ the turning point is a point of inflexion for $f(I_V^*).$ The polynomial $f(I_V^*)$ has only one endemic equilibrium.

For $\triangle >0$, we consider two cases; $a_1<0$ and $a_1>0$. If $a_1<0$, then $a_1a_3>0$. This means that $\sqrt{\triangle }<a_2$. Irrespective of the sign of $a_2$, $f'(I_V^*)$ has two real positive and distinct roots. This implies that (5) has two positive turning points. If $f(0) = a_0>0$ i.e $\mathcal {R}_0>1$ then, $f(I_V^*)$ has at least one positive real root, and hence at least one endemic equilibrium. On the other hand, if $f(0) = a_0<0$ then, $f(I_V^*)$ has at most two positive real roots when $\mathcal {R}_0<1$, and hence at most two endemic equilibria.

If $a_1>0$, then $a_1a_3<0$, which implies that $\sqrt{\triangle }>a_2$. For $a_2>0$, $f'(I_V^*)$ has two real roots of opposite signs. Since $f(0) = a_0>0$ for $\mathcal{R}_0>1$, then, $f(I_V^*)$ has one positive root. For $a_2<0$, $f'(I_V^*)$ has two negative real roots. Since $f(0) = a_0<0$ for $\mathcal{R}_0<1$, then, $f(I_V^*)$ has no positive real roots, and consequently no endemic equilibria.

Furthermore, we can use the *Descartes’ Rule of Signs* [7] to explore the existence of endemic equilibrium (or equilibria) for $\mathcal{R}_0<1$. We note the possible existence of backward bifurcation. The theorem below summarises the existence of endemic equilibria of the model system (2).

##### **Theorem 2**

*The model system* (2) has

1. *a unique endemic equilibrium point if*$\mathcal{R}_0>1$.
2. *has two endemic equilibria for*$\mathcal{R}_0^c<\mathcal{R}_0<1$*where*$\mathcal{R}_0^c$*is the critical threshold below which no endemic equilibrium exists.*

*Remark* The evaluation of $\mathcal{R}_0^c$ depends on the signs of $a_2$ and $a_1$ and the sign of the discriminant. The computations are algebraically involving and long and are not included here. Since the model system (2) possesses two endemic equilibria when $\mathcal{R}_0^c<\mathcal{R}_0<1$, the model exhibits backward bifurcation for $\mathcal{R}_0<1$.

The consequence of the above remark is that bringing $\mathcal{R}_0$ below unity is not sufficient to eradicate the disease. For eradication, $\mathcal{R}_0$ must be brought below the critical value $\mathcal{R}_0^c$.

#### Global stability of the endemic equilibrium

##### **Theorem 3**

*The endemic equilibrium point*$\mathbf{\mathcal {E}_1}$*of system* (2), *is globally asymptotically stable*.

##### *Proof*

The global stability of the endemic equilibrium, can be determined by constructing a Lyapunov function $\mathcal{V}(t)$ such that

9 $$\begin{aligned} \mathcal{V}(t)&= S_H -S_{H}^{*}-S_{H}^{*}\ln \frac{S_H}{S_{H}^{*}} +A\left( I_H -I_{H}^{*}-I_{H}^{*}\ln \frac{I_H}{I_{H}^{*}}\right) + B\left( I_V -I_{V}^{*}-I_{V}^{*}\ln \frac{I_V}{I_{V}^{*}}\right) \nonumber \\&\quad +C\left( I_{F} -I_{F}^{*}-I_{F}^{*}\ln \frac{I_{F}}{I_{F}^{*}}\right) + D\left( U -U^{*}-U^{*}\ln \frac{U}{U^{*}}\right) . \end{aligned}$$

The corresponding time derivative of $\mathcal{V}(t)$ is given by

10 $$\begin{aligned} \dot{\mathcal{V}}&= \left( 1 - \frac{S_{H}^{*}}{S_{H}}\right) \dot{S}_{H} + A\left( 1 - \frac{I_{H}^{*}}{I_{H}}\right) \dot{I}_{H} + B\left( 1 - \frac{I_{V}^{*}}{I_{V}}\right) \dot{I}_{V} \nonumber \\&\quad + C\left( 1 - \frac{I_{F}^{*}}{I_{F}}\right) \dot{I}_{F}+D\left( 1 - \frac{U^{*}}{U}\right) \dot{U}. \end{aligned}$$

At the endemic equilibrium, we have the following relations

11 $$\begin{aligned} \begin{array}{rcl} \mu _h+\theta _h&{}=&{} (\mu _h+\theta _h)S^{*}_{H} +\theta _h{I^*}_H+ m_1\beta _hS^{*}_{H}I^{*}_{V},\\ \mu _h+\gamma _h &{}=&{}m_1\beta _h\frac{S^{*}_{H}I^{*}_{V}}{{I^*}_H},\\ 1&{} =&{} m_2\beta _v\left( 1-I^{*}_{V}\right) \frac{I^{*}_{F}}{{I^*}_V}+ \eta _v\beta _v\left( 1-I^{*}_{V}\right) \frac{U^*}{{I^*}_V},\\ \mu _f&{} =&{} m_3\beta _f\left( 1-I^{*}_{F}\right) \frac{{I^*}_V}{I^{*}_{F}}+\eta _f\beta _f\left( 1-I^{*}_{F}\right) \frac{U^*}{I^{*}_{F}},\\ \mu _e&{} =&{} m_4\sigma _f\frac{I^{*}_{F}}{{U^*}}+m_5\sigma _v\frac{I^{*}_{V}}{{U^*}}. \end{array} \end{aligned}$$

Evaluating the components of the time derivative of the Lyapunov function using the relations (11) we have

12 $$\begin{aligned} \dot{\mathcal{V}}&= \left( 1 - \frac{S_{H}^{*}}{S_{H}}\right) \left[ (\mu _h+\theta _h)S_{H}^{*}\left( 1 - \frac{S_{H}}{S_{H}^{*}}\right) +\theta _h I_{H}^{*}\left( 1 - \frac{I_{H}}{I_{H}^{*}}\right) +m_1\beta _h S_{H}^{*}I_{V}^{*}\left( 1 - \frac{S_{H}I_{V}}{S_{H}^{*}I_{V}^{*}}\right) \right] \nonumber \\&\quad \quad + A\left( 1 - \frac{I_{H}^{*}}{I_{H}}\right) \left[ m_1\beta _h S_{H}^{*}I_{V}^{*}\left( \frac{S_{H}I_{V}}{S_{H}^{*}I_{V}^{*}}-\frac{I_{H}}{I_{H}^{*}}\right) \right] + B\left( 1 - \frac{I_{V}^{*}}{I_{V}}\right) \left[ m_2\beta _vI_{F}^{*}\left( \frac{I_{F}}{I_{F}^{*}}-\frac{I_{V}}{I_{V}^{*}}\right) \right. \nonumber \\&\quad \quad +\left. m_2\beta _vI_{F}^{*}I_V\left( 1-\frac{I_{F}}{I_{F}^{*}}\right) +\eta _v\beta _v U^{*}\left( \frac{U}{U^{*}}-\frac{I_{V}}{I_{V}^{*}}\right) +\eta _v\beta _vU^{*}I_V \left( 1-\frac{U}{U^{*}}\right) \right] \nonumber \\&\quad \quad + C\left( 1 - \frac{I_{F}^{*}}{I_{F}}\right) \left[ \eta _f\beta _fU^{*}\left( \frac{U}{U^{*}}-\frac{I_{F}}{I_{F}^{*}}\right) +\eta _f\beta _fU^{*}I_F\left( 1-\frac{U}{U^{*}}\right) \right. \nonumber \\&\quad \quad +\left. m_3\beta _fI_{V}^{*}\left( \frac{I_{V}}{I_{V}^{*}}-\frac{I_{F}}{I_{F}^{*}}\right) +m_3\beta _fI_{V}^{*}I_F\left( 1-\frac{I_{V}}{I_{V}^{*}}\right) \right] \nonumber \\&\quad \quad +D\left( 1 - \frac{U^{*}}{U}\right) \left[ m_4\sigma _f{I_F}^{*}\left( \frac{I_{F}}{I_{F}^{*}}-\frac{U}{U^{*}}\right) +m_5\sigma _v{I_V}^{*}\left( \frac{I_{V}}{I_{V}^{*}}-\frac{U}{U^{*}}\right) \right] . \end{aligned}$$

Let

13 $$\begin{aligned} v=\frac{S_H}{S^{*}_{H}},&w=\frac{I_H}{I^{*}_{H}}, x=\frac{I_V}{I^{*}_{V}},y=\frac{I_F}{I^{*}_{F}}\quad \mathrm{and}\quad z=\frac{U}{U^{*}}. \end{aligned}$$

Substituting (13) into (12), we obtain

14 $$\begin{aligned} \dot{\mathcal{V}}= & {} -(\mu _h+\theta _h)S_{H}^{*}\frac{( 1 - v)^2}{v}+\mathcal{H}(v,w,x,y,z), \end{aligned}$$

where

15 $$\begin{aligned} \mathcal{H}&= \theta _h I_{H}^{*}\left( 1 -w-\frac{1}{v}+\frac{w}{v}\right) +m_1\beta _h S_{H}^{*}I_{V}^{*}\left( 1 - \frac{1}{v}+x-xv\right) \nonumber \\&\quad \quad + A m_1\beta _h S_{H}^{*}I_{V}^{*}\left( 1+xv-w-\frac{vx}{w}\right) + B m_2\beta _vI_{F}^{*}\left( 1+y-x-\frac{x}{y}\right) \nonumber \\&\quad \quad +B m_2\beta _vI_{F}^{*}{I^*}_V\left( x+y-xy-1\right) +B\eta _v\beta _v U^{*}\left( 1+z-x-\frac{z}{x}\right) \nonumber \\&\quad \quad +B\eta _v\beta _vU^{*}{I^*}_V \left( x+z-xz-1\right) + Cm_3\beta _f{I_V}^{*}\left( 1+x-y-\frac{x}{y}\right) \nonumber \\&\quad \quad + Cm_3\beta _f{I_V}^{*}{I_F}^{*}\left( y+x-xy-1\right) +C\eta _f\beta _fU^{*}\left( 1+z-y-\frac{z}{y}\right) \nonumber \\&\quad \quad +C\eta _f\beta _fU^{*}{I_F}^*\left( y+z-yz-1\right) +Dm_4\sigma _f{I_F}^{*}\left( 1+y-z-\frac{y}{z}\right) \nonumber \\&\quad \quad +Dm_5\sigma _v{I_V}^{*}\left( 1+x-z-\frac{x}{z}\right) . \end{aligned}$$

Next, we choose *A*, *B*, *C* and D so that none of the variable terms of $\mathcal{H}$ are positive. It is important to group together the terms in $\mathcal{H}$ that involve the same state variable terms, as well as grouping all of the constant terms together. So we can show that $\mathcal{H}<0$ by expanding (15), writing out the constant term and the coefficients of the variable terms such as $v,w,x,y,z,\frac{1}{v},\frac{w}{v},\frac{x}{v}$ and so on. The only variable terms that appear with positive coefficients are *x*, *y* and *z*. We thus choose the Lyapunov coefficients so as to make the coefficients of*x*, *y* and *z* equal to zero. We have

$$\begin{aligned} A&=1, B=\frac{m_1\beta _hS_{H}^{*}I_{V}^{*}}{m_2\beta _vI_{V}^{*}(1-I_{F}^{*})+\eta \beta _vU^{*}(1-I_{V}^{*})}. \end{aligned}$$

The coefficients *C* and *D* can similarly be evaluated from the coefficients of *y* and *z*. Note that expressions such as

$$\begin{aligned} m_1\beta _hS_{H}^{*}I_{V}^{*}\left( 2-\frac{1}{v}-\frac{xv}{w}\right) \end{aligned}$$

emanating from the substitution of the coefficients into $\mathcal{H},$ are less than or equal to zero by the arithmetic mean-geometric mean inequality. This implies that $\mathcal{H}\le 0$ with equality only if $\frac{S_H}{{S_H}^*}=\frac{I_H}{{I_H}^*} = \frac{I_V}{{I_V}^*}=\frac{I_F}{{I_F}^*}=\frac{U}{{U}^*}=1.$

Therefore, $\dot{\mathcal{V}} \le 0$ and by the LaSalle’s Extension [17], it implies that the omega limit set of each solution lies in an invariant set contained in ${\Omega }.$ The only invariant set contained in $\Omega$ is the singleton $\mathcal{E}_1$. This shows that each solution which intersects $\mathbb {R}_+^5$ limits to the endemic equilibrium. This completes the proof. $\square$

## Results

### Parameter estimation

The biggest challenge in epidemic modeling is the estimation of parameters in the model validation process. In this section we endeavour to estimate some of the parameter values of system (2). The demographic parameters can be easily estimated from census population data. We begin by estimating the mortality rate $\mu _h.$ We note that the average life expectancy of the human population in Ghana is 60 years [21]. This translates into $\mu _h=0.017$ per year or equivalently $4.6\times 10^{-5}$ per day. Buruli ulcer is currently regarded as a vector borne disease. Recovery rates modelled by $\gamma _h,$ of vector borne diseases range from $1.6\times 10^{-5}$ to 0.5 per day [23]. This translates to an average of between 0.00584 and 183 per year. The rate of loss of immunity $\theta _h$ for vector borne diseases range between 0 and $1.1\times 10^{-2}$ per day[23]. The mortality rate of the water bugs is assumed to be 0.15 per day [3]. The rates per day can easily be transferred to yearly rates.

In this model we shall assume that we have more water bugs than humans so that $m_1<1.$ Since the water bugs prey on the fish, a reasonable food chain structure leads to the assumption that we have more fish than water bugs hence $m_2>1$ and consequently $0<m_3<1.$ If the water bug is assumed to interact more with the environment than fish then $\eta _v >1$ and $0<\eta _f<1.$ The natural mortality of small fish in rivers is not well documented and data on the mortality of river fish in Ghana is not available. For the purpose of our simulations, we shall assume that $3\times 10^{-3}<\mu _f<7\times 10^{-3}$ per day. Given that $K\ge N_F,N_V$ we have $0\le m_4,m_5\le 1.$ We shall also assume that $0\le \sigma _f,\sigma _v\le 1.$ We summarise the parameters in the following Table 2.

**Table 2:** Parameter values used for the simulations and sensitivity analysis

Parameter	Value/range	Source
$\mu _h$	$4.5\times 10^{-5}$	[21]
$\gamma _h$	$1.6\times 10^{-5}-0.5$	[23]
$\theta _h$	$0-1.1\times 10^{-2}$	[23]
$m_1,m_2$	$m_1<1,m_2>1$	Estimated
$m_3,m_4,m_5$	(0,1)	Estimated
$\beta _h,\beta _f,\beta _v$	(0,1)	Estimated
${\eta _v}$	(1,5)	Estimated
${\eta _f}$	(0, 1)	Estimated
${\sigma _f,\sigma _v}$	(0,1)	Estimated
${\mu _f}$	$3\times 10^{-3}-7\times 10^{-3}$	Estimated
$\mu _e$	(0,1)	Estimated

### Sensitivity analysis

**Fig. 2:**
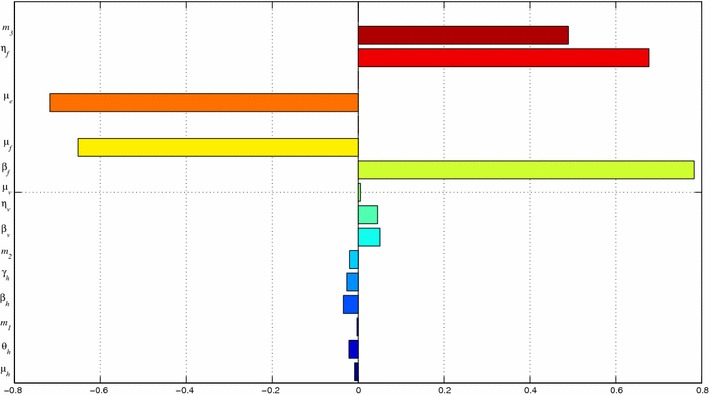
PRCC plots: The variable *U* largely depends on $m_3,~ \eta _f,~\mu _e,~\mu _f$ and $\beta _f$. The *bars* pointing to the *left* indicate that *U* has an inverse dependence on the respective parameters. We observe that the parameters $m_3,~ \eta _f$ and $\beta _f$ aggravate the disease when they are increased while $\mu _f$ and $\mu _e$ reduce its severity when increased

Many of the parameters used in this paper are not determined experimentally. Therefore their accuracy is always in doubt. This can be overcome by observing responses of such parameters and their influence on the model variables through sensitivity and uncertainty analysis. In this subsection we present the sensitivity analysis of the model parameters to ascertain the degree to which the parameters affect the outputs of the model. We use the Partial Correlation Coefficients (PRCCs) analysis to determine the sensitivity of our model to each of the parameters used in the model. Through correlations, the association of the parameters and state variables can be established. In our case, we determine the correlation of our parameters and the state variable *U*. Alongside the PRCCs are the statistical significance test p-values for each of the parameters. If the magnitude of the PRCC value of a parameter is greater than 0.5 or less than −0.5 and the p-value less than 0.05, then the model is sensitive to the parameter. On the other hand, PRCC values close to $+1$ or $-1$ indicate that the parameter strongly influences the state variable output. The sign of a PRCC value indicates the qualitative relationship between the parameter and the output variable. A negative sign indicates that the parameter is inversely proportional to the outcome measure [10]. The parameters with negative PRCCs reduce the severity of Burili ulcer disease while those with positive PRCCs aggravate it. Using Latin Hypercube Sampling (LHS) scheme with 1000 simulations for each run, with *U* as the outcome variable. Our results show that the variable *U* is sensitive to the changes in the parameters $m_3,~ \eta _f,~\mu _e,~\mu _f$ and $\beta _f$. The results are shown in Fig. 2.

The results from the PRCC analysis are summarized in Table 3. The significant parameters together with their PRCC values and p-values have been encircled.

**Table 3:**
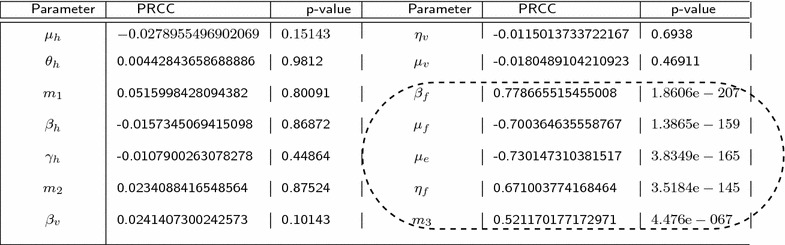
Outputs from PRCC analysis

**Fig. 3:**
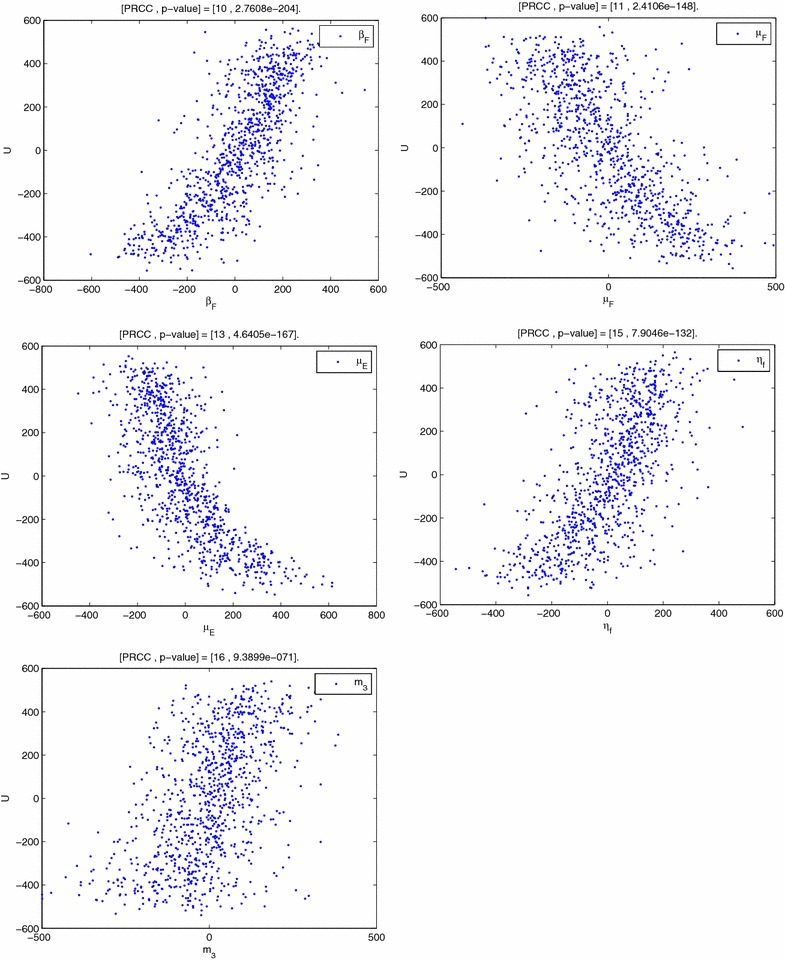
PRCC plots shows the PRCC plots for the parameters $\beta _f$, $\mu _f$, $\mu _e$, $\eta _f$ and $m_3$

In Fig. 3 the residuals for the ranked Latin Hypercube Sampling parameter values are plotted against the residuals for the ranked density of *Mycobacterium ulcerans*.The PRCC plots for parameters $\beta _f,~\mu _f,~\mu _e$ and $\eta _f$ show a strong linear correlation. The growth of *Mycobacterium ulcerans* increases as the number of infected fish that eventually shed bacteria into the environment increases. An increase in the parameters $\mu _f$ and $\mu _e$ leads a decrease in amount of bacteria in the environment.

### Data and the fitting process

One of the most important steps in the model building chronology is model validation. We now focus on the data provided by the Ashanti Regional Disease Control Office for Buruli ulcer cases in Ghana per 10,0000 people. The data are given in the Table 4 below for the years 2003–2012.

**Table 4:** Data on Buruli ulcer cases in Ghana

Year	2003	2004	2005	2006	2007	2008	2009	2010	2011	2012
Buruli ulcer cases	739	1159	1201	1096	1136	1300	1158	1428	1324	1292

Source [13]

We fit the model system (2) to the data of Buruli ulcer cases expressed as fractions. We use the least squares curve fit routine (lsqcurvefit) in Matlab with optimisation to estimate the parameter values. Many parameters are known to lie within limits. A few parameters such as the demographic parameters are known [13] and it is thus important to estimate the others. The process of estimating the parameters aims at finding the best concordance between computed and observed data. One tedious way to do it is by trial and error or by the use of software programs designed to find parameters that give the best fit. Here, the fitting process involves the use of the least squares-curve fitting method. Matlab code is used where unknown parameter values are given a lower and upper bound from which the set of parameter values that produce the best fit are obtained.

**Fig. 4:**
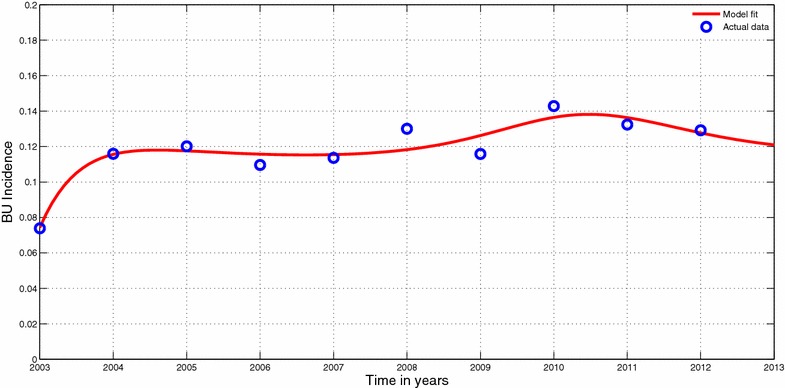
Model fit to data. Model system (2) fitted to data of Burili ulcer cases in Ghana. The *circles* indicate the actual data and the *solid line* indicates the model fit to the data. The parameter values used for the fitting; $\mu _h=0.000045,~\theta =0.1,~m_1=5,~\beta _h=0.1,~\gamma =0.056,~m_2=10,~\beta _v=0.000065,~\eta _v=1.5,~\eta _f=0.6,~\mu _V=0.15,~\beta _f=0.00005,~\mu _f=0.05,~\sigma _f=0.05,~\sigma _v=0.006,~\mu _e=0.4$

Figure 4 shows how system (2) fits to the available data on the incidence of the BU. The incidence solution curve shows a very reasonable fit to the data.

**Fig. 5:**
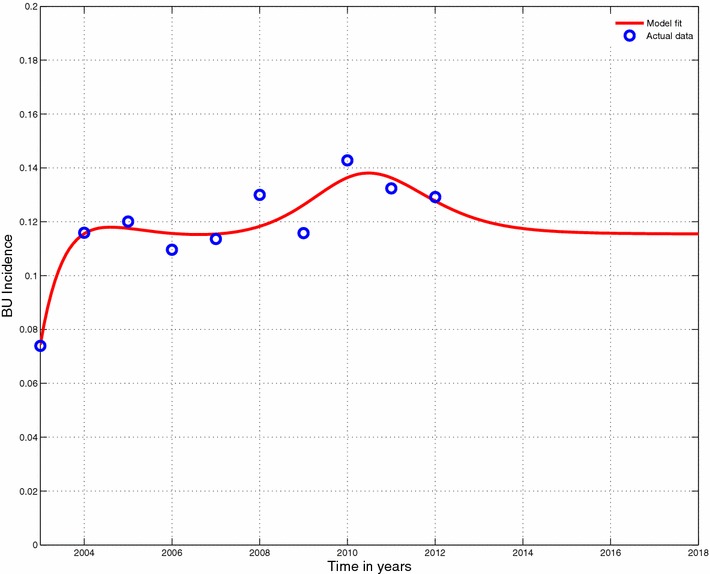
Projected model fit. Projection to fit in Fig. 4

In planning for a long term response to the Buruli ulcer epidemic, it is important to have some reasonable projections to the epidemic. The fitting process allows us to envisage the Buruli ulcer epidemic in future. it is important to note that the projections are reasonably good over a short period of time since the current is evolving gradually based on the available data. We chose to project the epidemic beyond 5 years to 2017. Figure 5 show the projected Buruli ulcer epidemic.

**Fig. 6:**
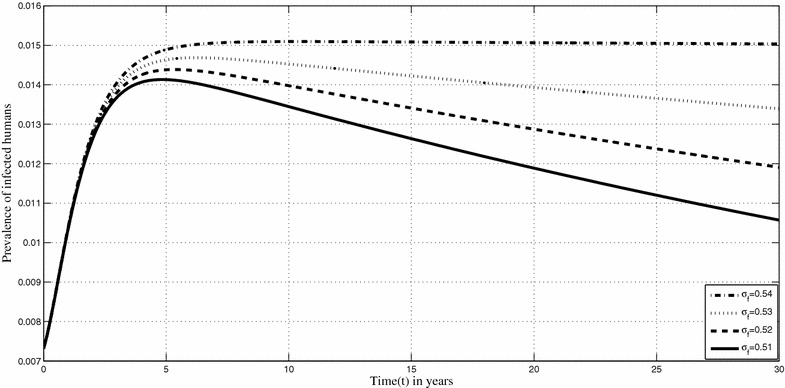
Prevalence of Buruli ulcer infection in humans. Shows prevalence humans when $\sigma _f$ is varied

**Fig. 7:**
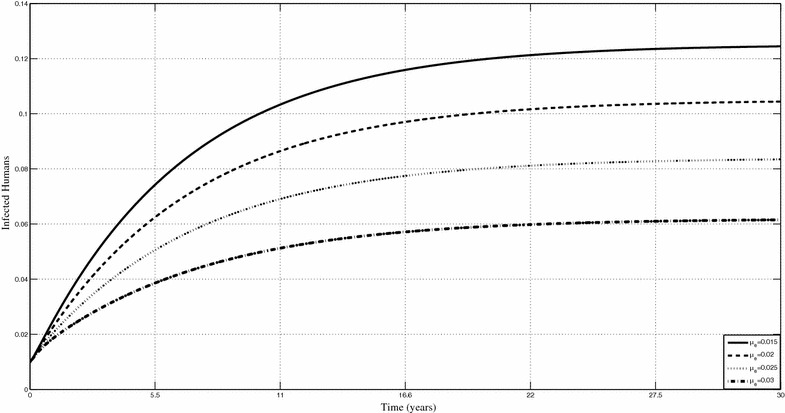
Prevalence of Buruli ulcer in infected humans for different values of ${\mu }_{e.}$ Shows prevalence the infected humans when $\mu _e$ is varied

Figures 6 and 7 show the changes in the prevalence of infected humans respectively when $\sigma _f,$ the shedding rate of *Mycobacterium ulcerans* in the environment and $\mu _e$ the removal rate of MU from the environment, are varied. Based on the sensitivity analysis, our model is very sensitive to the shedding rate of *Mycobacterium ulcerans* into the environment. Figure 6 shows that an increase in the shedding rate will lead increased human infections. We can actually quantify the related increases. For instance, if $\sigma _f$ is increased from 0.51 to 0.52 on year 15, the percentage increase in the prevalence of human infections is 6 %. Minimising *Mycobacterium ulcerans*in the environment is an important control measure that is, albeit impractical at the moment. We observe through our results that their decrease in the environment can lead to quantifiable changes in the prevalence of infected humans. Increasing $\mu _e$ leads to a decrease in the prevalence of infected humans.

## Discussion

In this paper, a deterministic model on the dynamics of the Buruli ulcer in the presence of a preventive intervention strategy is presented. The model’s steady states are determined and their stabilities investigated in terms of the classic threshold $\mathcal{R}_0.$ In disease transmission modelling, it is well known that a classical necessary condition for disease eradication is that the basic reproductive number $\mathcal{R}_0,$ must be less than unity. The model has multiple endemic equilibria (in fact it exhibits a backward bifurcation). When a backward bifurcation occurs, endemic equilibria coexist with the disease free equilibrium for $\mathcal{R}_0<1.$ This means that getting the classic threshold $\mathcal{R}_0$ less than 1, might not be sufficient to eliminate the disease. Thus the existence of backward bifurcation has important public health implications. This might explain why the disease has persisted in the human population over time. The endemic equilibrium is found to be globally stable if $\mathcal{R}_0>1.$

The sensitivity analysis of model parameters showed some interesting results. These results suggest that efforts to remove *Mycobacterium ulcerans* and infected fish from the environment will greatly reduce the epidemic although the latter will be impracticable. This is because of the costs involved and the fact that many governments in affected areas operate on lean budgets.

The model is then fitted to data on the Buruli ulcer in Ghana. The model reasonably fits the data. The challenge in the fitting process was that the data appears to indicate that Buruli ulcer has reached a steady state. This then produced some parameter values that appeared unreasonable. Despite these challenges, the fit produced reasonable projections on the future of the ulcer. The model shows that in the near future, the number of cases will not change if everything remains the same. An important consideration that can be added to the model is the inclusion of probable policy shifts and the investigation of different scenarios on the progression of the epidemic as the policies change. Because not much of the disease is understood, parameter estimation was a daunting task. So we had to reasonably estimate some of the parameter using the hypothesis that Buruli ulcer is a vector borne disease. Due to the estimation of essential parameters sensitivity analysis was necessary and very important to determine how these parameters influence the model. The implications of varying some of the important epidemiological parameters such as the shedding rates were investigated. Important results were drawn through Figs. 6 and 7. The main result of this paper is that the management of Buruli ulcer depends mostly on the management of the environment.

## Conclusions

This model can be improved by considering social interventions in the human population, modeled as functions and the inclusion of the different forms of treatment available as some individuals opt for traditional methods while others depend on the government health care system [1]. Social interventions include education, awareness, poverty reduction and provision of social services. While the mathematical representations of these interventions are insurmountable, they are vital to the dynamics of the disease and public health policy designs. Finally this model can be used to suggest the type of data that should be collected as research on the Buruli ulcer intensifies. The global burden of the disease and its epidemiology are not well understood, [28]. Clearly, gaps do exist in the nature and type of data available. Reports on the disease are often based on passive presentations of patients at health care facilities. As a result of the difficulties of accessing health care in affected areas, data on the disease is scanty.
